# SARS-CoV-2 RdRp Inhibitors Selected from a Cell-Based SARS-CoV-2 RdRp Activity Assay System

**DOI:** 10.3390/biomedicines9080996

**Published:** 2021-08-11

**Authors:** Jung Sun Min, Sunoh Kwon, Young-Hee Jin

**Affiliations:** 1 KM Convergence Research Division, Korea Institute of Oriental Medicine, Daejeon 34054, Korea; jsmin1019@kiom.re.kr; 2 Center for Convergent Research of Emerging Virus Infection, Korea Research Institute of Chemical Technology, Daejeon 34114, Korea; 3 KM Application Center, Korea Institute of Oriental Medicine, Daegu 41062, Korea

**Keywords:** COVID-19, SARS-CoV-2, RNA-dependent RNA polymerase, RdRp, remdesivir, lycorine, cepharanthine, adefovir dipivoxil, emtricitabine, telbivudine, entecavir hydrate, moroxydine, rifampin, therapeutics

## Abstract

The COVID-19 pandemic, caused by severe acute respiratory syndrome coronavirus 2 (SARS-CoV-2), urgently needs effective prophylactic and therapeutic drugs. RNA-dependent RNA polymerase (RdRp), essential for replicating and transcribing a viral RNA genome, is highly conserved in coronaviruses; thus, it is a potential target for inhibiting coronavirus infection. In this study, we generated the cell-based SARS-CoV-2 RdRp activity assay system by modifying a previously reported cell-based MERS-CoV RdRp activity assay system to screen for SARS-CoV-2 RdRp inhibitors. The assay system consisted of an expression plasmid encoding SARS-CoV-2 RdRp and an RdRp activity reporter plasmid. RdRp activity in the cells could be conveniently detected by luminescence after transfection. We confirmed that SARS-CoV-2 RdRp replicated double-stranded RNA using immunofluorescence staining and the inhibition of RdRp activity by remdesivir and lycorine using this system. Moreover, the Z-factor of this system was calculated to be 0.798, suggesting the reproducibility and reliability of the high-throughput screening system. Finally, we screened nucleoside and nucleotide analogs and identified adefovir dipivoxil, emtricitabine, telbivudine, entecavir hydrate, moroxydine and rifampin as novel SARS-CoV-2 RdRp inhibitors and therapeutic candidates for COVID-19 This system provides an effective high-throughput screening system platform for developing potential prophylactic and therapeutic drugs for COVID-19 and emerging coronavirus infections.

## 1. Introduction

The coronavirus disease 2019 (COVID-19) pandemic, caused by severe acute respiratory syndrome coronavirus 2 (SARS-CoV-2), broke out in December 2019. As of July 2021, COVID-19 has resulted in approximately 190 million confirmed cases with a 2% death rate, according to the World Health Organization [1].

Like other emerging coronaviruses, such as MERS-CoV and SARS-CoV, SARS-CoV-2 belongs to the *betacoronavirus* genus. SARS-CoV-2 has a positive-sense, single-stranded 30-kb RNA genome. The homology of the SARS-CoV-2 genome to SARS-CoV and MERS-CoV is around 80% and 50%, respectively [2]. The SARS-CoV-2 genome encodes pp1a and pp1ab polypeptides from open reading frame (ORF)1a and ORF1b, respectively and four structural proteins including spike, envelope, membrane and nuclocapsid with intervening ORF encoding six accessory proteins [3]. The pp1a and pp1ab are cleaved by self-proteolytic cleavage mediated by viral proteases into 16 nonstructural proteins (nsps) [4,5].

Among the viral nsps, nsp12 harboring the RNA-dependent RNA polymerase (RdRp) is an essential enzyme for mediating the replication and transcription of the viral genome [6]. Since SARS-CoV-2 RdRp is 96% homologous to the SARS-CoV RdRp and 70% to the MERS-CoV RdRp, the genomic sequences encoding RdRp are highly conserved among the emerging coronaviruses [7]. Among the SARS-CoV-2 variants, the spontaneous mutations have been rarely observed in the RdRp region compared to that in the region encoding the spike protein [8]. Thus, SARS-CoV-2 RdRp is a potential target for prophylactic and therapeutic drugs for treating patients with COVID-19 induced by the many SARS-CoV-2 variants.

Remdesivir is known to target SARS-CoV-2 RdRp. It mimics an RNA nucleotide building block and covalently incorporates it into the replicating strand, thus terminating RNA chain elongation mediated by the SARS-CoV-2 RdRp complex [9]. As an FDA-approved first-in-class drug for COVID, remdesivir has been extensively utilized for treating COVID-19 patients. However, to achieve better efficacy against COVID-19, effective prophylactic and best-in-class therapeutic drugs are under development.

We need to establish a high-throughput screening (HTS) platform to measure RdRp activity of RNA synthesis quantitatively to identify potent SARS-CoV-2 RdRp inhibitor candidates. Cell-free enzymatic assay systems are usually used to measure RdRp activity [9,10]. While high-purity functionally active recombinant RdRp, RNA templates, RNA primers and radioisotope-labeled NTPs can be prepared, it is challenging to optimize the reaction conditions and the method of detecting dsRNA products [11]. Moreover, it is difficult to quantify RdRp activity and screen for RdRp inhibitors in an HTS system.

In this study, we generated a cell-based SARS-CoV-2 RdRp activity assay system by modifying a previously established cell-based assay system for MERS-CoV RdRp activities [12]. In addition, we validated this assay system and its application to the screening of SARS-CoV-2 RdRp inhibitors. This system could be the HTS platform for identifying SARS-CoV-2 RdRp inhibitors and developing them into prophylactic or therapeutic drugs for COVID-19.

## 2. Materials and Methods

### 2.1. Test Compounds

Remdesivir (PubChem CID: 121304016) (MedchemExpress, Princeton, NJ, USA), lycorine hydrochloride (PubChem CID: 164943) and cepharanthine (PubChem CID: 10206) (Wuhan ChemFaces Biochemical, Wuhan, China) were purchased. Additionally, nucleoside or nucleotide analogs adefovir dipivoxil (PubChem CID: 60871), emtricitabine (PubChem CID: 60877), telbivudine (PubChem CID: 159269), entecavir hydrate (PubChem CID: 135526609), moroxydine HCl (PubChem CID: 76621) and rifampin (PubChem CID: 135398735) (Selleckchem, Pittsburgh, PA, USA) were procured; the10 mM stock solution of each analog in dimethyl sulfoxide (Sigma-Aldrich, Burlington, MA, USA) was stored at −80 °C.

### 2.2. Plasmid Construction

SARS-CoV nsp12 gene (GenBank Accession No. YP_009725307) with an N-terminal (N-term) or C-terminal (C-term) Flag, nsp7 gene (GenBank Accession No. YP_009725303) with or without a C-term Flag, nsp8 gene (GenBank Accession No. YP_009725304) with or without a C-term Flag were human codon-optimized and synthesized (GENEWIZ, South Plainfield, NJ, USA). The genes were then cloned between the NheI and XhoI restriction enzyme sites within the multiple cloning region of the pCI plasmid (Promega Corporation, Madison, WI, USA) to construct pCI-SARS-CoV-2 nsp12-N-term Flag (pCI-SARS2-nsp12N), pCI-SARS-CoV-2 nsp12-C-term Flag (pCI-SARS2-nsp12C), pCI-SARS-CoV-2 nsp7-C-term Flag (pCI-SARS2-nsp7C), pCI-SARS-CoV-2 nsp7 (pCI-SARS2-nsp7), pCI-SARS-CoV-2 nsp8-C-term Flag (pCI-SARS2-nsp8C), pCI-SARS-CoV-2 nsp8 (pCI-SARS2-nsp8) and pCI-SARS-CoV-2 nsp8-IRES-nsp7 (pCI-SARS2-nsp8-IRES-nsp7). The N-terminal Flag-tagged nsp5/3CLpro (GenBank Accession No. YP_009725301), optimized to human codons, was inserted between the NheI and XhoI sites within multiple cloning sites of pcDNA3.1(+) to create pcDNA3.1-SARS2-nsp5N.

**Figure 1:**
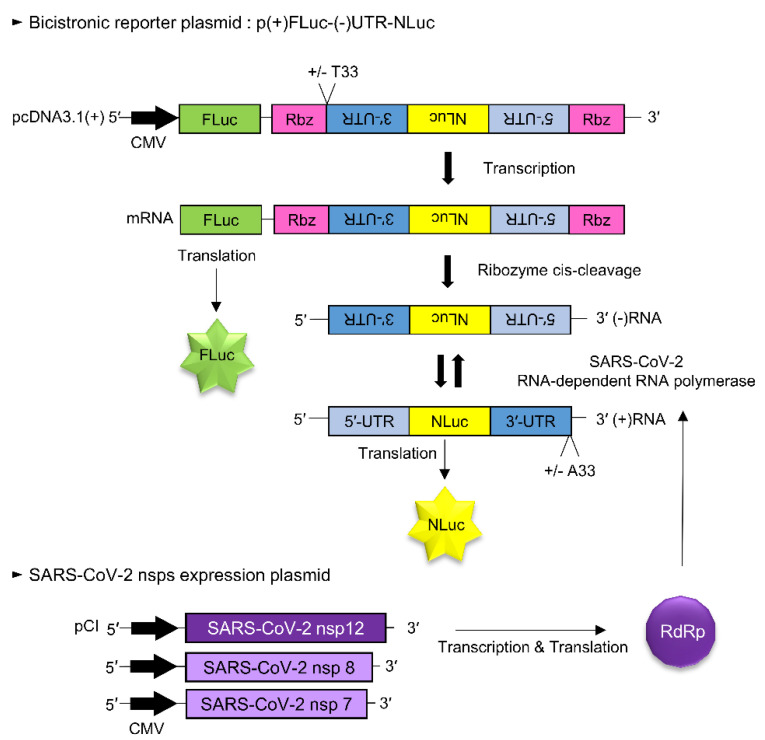
Schematic diagram of the cell-based SARS-CoV-2 RdRp activity assay system. A SARS-CoV-2 RdRp reporter assay system consists of a bicistronic reporter plasmid and nsp12 gene expression plasmid. A p(+)FLuc-(−)UTR-NLuc reporter plasmid contained the sense-oriented (+) firefly luciferase gene, (+)FLuc under the CMV promoter and the antisense-oriented 3′-UTR of SARS-CoV-2, Nano-Glo luciferase sequence and 5′-UTR of SARS-CoV-2, (−)UTR-NLuc, which were flanked with HDV ribozyme self-cleavage sequences. The host RNA polymerase transcribed the (+)FLuc-(−)UTR-NLuc RNA, which was then processed at the HDV ribozyme self-cleavage sequence. The cleaved (−)3′-UTR-(−)NLuc-(−)5′-UTR RNA sequences were replicated by the SARS-CoV RdRp protein. Then, the replicated sense-oriented NLuc RNA was translated. Therefore, NLuc activity indicated the activity of SARS-CoV-2 RdRp, whereas FLuc activity acted as an internal control.

We generated the reporter plasmid by modifying a previously published bicistronic MERS-CoV RdRp reporter construct [12]. Then, the sense-oriented (+) firefly luciferase gene (FLuc) was cloned between the NheI and HindIII sites within the multiple cloning sites of pcDNA3.1(+). Subsequently, the hepatitis delta virus (HDV) ribozyme sequence, antisense (−) 3′-untranslated region (UTR) of SARS-CoV-2, antisense Nano-Glo^®^ luciferase gene (NLuc) (Promega, GenBank Accession No. KM359770), antisense 5′-UTR of SARS-CoV-2 and HDV ribozyme sequence were sequentially synthesized (GENEWIZ) and cloned between the HindIII and XhoI sites downstream of the (+)FLuc gene to generate pcDNA3.1-(+)FLuc-(−)UTR-NLuc reporter (p-(+)FLuc-(−)UTR-NLuc) (Figure 1).

### 2.3. Cells and Transfection

HEK293T cells (30 passages, American Type Culture Collection, Manassas, VA, USA) were cultured in Dulbecco’s Modified Eagle’s Medium (Corning Inc., Corning, NY, USA) supplemented with 10% fetal bovine serum (Gibco, Carlsbad, CA, USA) and 1% penicillin/streptomycin (Gibco) at 37 °C in 5% CO_2_. In addition, transIT^®^-LT1 (Mirus Bio LLC., Madison, WI, USA) was used for the transient transfection.

### 2.4. Cell-Based SARS-CoV-2 RdRp Activity Assay

HEK293T cells were seeded in a 96-well plate and pCI-SARS-CoV-2 nsp12-N and p(+)FLuc-(−)UTR-NLuc reporter plasmids were transfected with or without the SARS-CoV-2 nsp7 and nsp8 expression plasmids. After 24 h, the FLuc and NLuc values of these cells were measured using the Nano-Glo^®^ Dual-Luciferase^®^ Reporter Assay System (Promega) and the NLuc values were normalized with FLuc.

### 2.5. Western Blot Assay

HEK293T cells were transfected with the indicated plasmids for 24 h and lysed in the Glo Lysis Buffer (Promega Corporation). The lysates were separated on a gradient gel (Bio-Rad Laboratories, Hercules, CA, USA) and transferred to a nitrocellulose membrane (Bio-Rad Laboratories). The membrane was incubated with antibodies against Flag (Cat no. ab125243, Lot no. GR3348594-1, Abcam, Cambridge, UK) or β-actin (Cat no. #3700, Lot no. 5, Cell Signaling Technology, Danvers, MA, USA), then with an HRP-conjugated secondary antibody (Cat no. ab6728, Lot no. GR3200472-2, Abcam) and detected with chemiluminescence substrates (Thermo Fisher Scientific, Waltham, MA, USA) using the ChemiDoc^TM^ Touch Imaging System (Bio-Rad).

### 2.6. Immunofluorescence Staining Assay

HEK293T cells were fixed with 4% paraformaldehyde and permeabilized with 0.2% Triton X-100 in PBS. After blocking with 3% bovine serum albumin, the cells were incubated with anti-double-stranded RNA (dsRNA) antibody K1 (Cat. No. 10020200, Batch no. K1-1715, Scicons, Susteren, the Netherlands) and then with the AlexaFluor555 conjugated anti-mouse immunoglobulin G (Thermo Fisher). The labeled cells were mounted on slides with the SlowFade Gold anti-fade reagent with DAPI (Invitrogen) and visualized by fluorescence microscopy (Olympus Corporation, Tokyo, Japan) and the CellSense program (Olympus).

### 2.7. Calculation of Z-Factor

Z-factors were calculated using the following equation [13]: Z-factor = 1 − [(3SD_Negative_ + 3SD_Positive_)/|mean_Negative_ − mean_Positive_|]. The negative group (n = 30 wells) indicated the dual transfection with p(+)FLuc-(−)UTR-NLuc and pCI (control) and the positive group (n = 30 wells) denoted the dual transfection with pcDNA3.1-(+)FLuc-(−)UTR-NLuc and pCI-SARS2 nsp12N.

### 2.8. Statistical Analysis

The data were presented as mean ± standard error of the mean. Statistical comparisons were conducted using one- or two-way analysis of variance (ANOVA) followed by Bonferroni’s multiple comparison test. The non-linear regression analysis of IC_50_ was conducted using GraphPad Prism^®^9.1.2 (GraphPad Software Inc., San Diego, CA, USA).

## 3. Results

### 3.1. Establishment of the Cell-Based SARS-CoV-2 RdRp Activity Assay System

We generated the SARS-CoV-2 RdRp activity assay system by modifying a previously reported cell-based MERS-CoV RdRp activity assay system [12]. First, we constructed various SARS-CoV-2 RdRp expression plasmids and RdRp activity reporter plasmids (Figure 1). The bicistronic reporter plasmid [p(+)FLuc-(−)UTR-NLuc] comprised a sense-oriented (+) firefly luciferase sequence [(+)FLuc] acting as an internal control and an antisense-oriented Nano-Glo luciferase sequence [(−)NLuc] flanked by HDV ribozyme self-cleavage sequences and an antisense 5′-UTR and 3′-UTR of SARS-CoV-2. The host cell’s DNA-dependent RNA polymerase transcribed the full-length (+)FLuc-(−)UTR-NLuc RNA, which was then cleaved at the HDV ribozyme self-cleavage sites. Next, the cleaved antisense 3′-UTR-(−)NLuc-antisense 5′-UTR RNA sequences were replicated by SARS-CoV RdRp encoded by the transfected expression plasmid. Finally, the replicated sense-oriented NLuc RNA was translated and the assayed activity of NLuc represented the SARS-CoV-2 RdRp activity.

**Figure 2:**
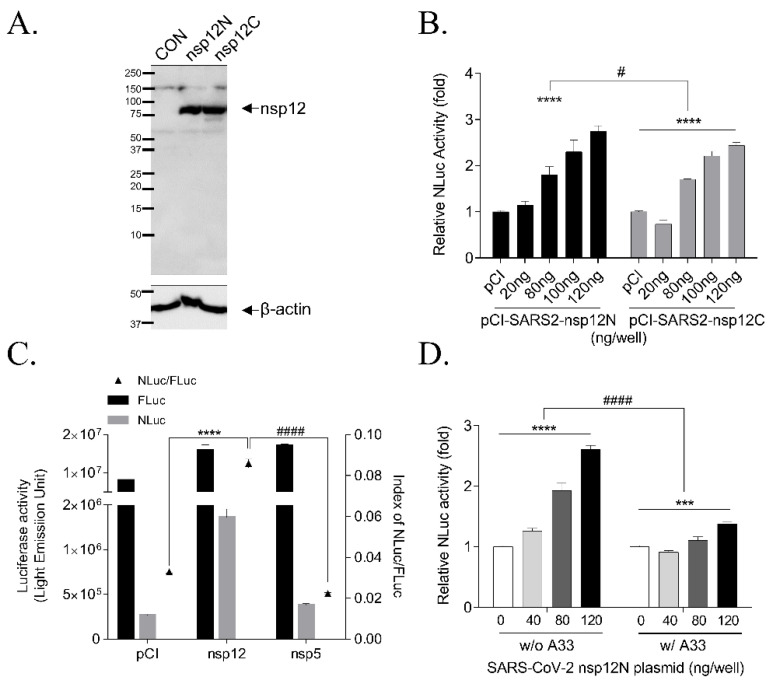
Expression of the SARS-CoV-2 RdRp gene and RdRp activity in a cell-based SARS-CoV-2 RdRp activity assay. (**A**) Expression of the SARS-CoV-2 RdRp gene with an N-terminal or C-terminal Flag was detected with an anti-Flag antibody by western blotting 24 h after transfection with pCI empty plasmid (CON), pCI-SARS2-nsp12N plasmid (nsp12N), or pCI-SARS2-nsp12C plasmid (nsp12C). (**B**) The activities of nsp12N and nsp12C were compared in a cell-based assay system by transfecting pCI-SARS2-nsp12N or pCI-SARS2-nsp12C and p(+)FLuc-(−)UTR-NLuc at the indicated concentrations for 24 h. The NLuc value was normalized to that of FLuc and analyzed by two-way ANOVA with Bonferroni’s multiple comparison tests [*n* = 3, **** *p* < 0.0001, plasmid dosage effect, F(4, 20) = 81.90; # *p* = 0.0205, Flag tag effect, F(1, 20) = 6.337; ns, *p* = 0.3754, Flag tag times plasmid dose interaction, F(4, 20) = 1.119]. (**C**) NLuc activity was increased by transfection with pCI-SARS2-nsp12N (nsp12) but not with pcDNA3.1-SARS2-nsp5N (nsp5). After FLuc and NLuc activities were measured, NLuc/FLuc values were graphed. The results were analyzed by one-way ANOVA with Bonferroni’s multiple comparison tests [*n* = 3, F(2, 6) = 672.2, **** *p* < 0.0001 vs. pCI, #### *p* < 0.0001 vs. nsp5]. (**D**) The p(+)FLuc-(−)UTR-NLuc reporter plasmids with or without polyA_33_ were compared by transfection with pCI-SARS2-nsp12N and p(+)FLuc-(−)UTR-NLuc without poly-A_33_ (w/o A_33_) or with poly-A_33_ (w/ A_33_) at the indicated concentrations. The relative NLuc activities (normalized by FLuc activity) of the two groups were compared using two-way ANOVA followed by Bonferroni’s multiple comparison [*n* = 3; **** *p* < 0.0001, dose-effect, F(3, 8) = 89.08; *** *p* = 0.0001, dose-effect F(3, 8) = 29.31; #### *p* < 0.0001; poly-A_33_ effect, F(1, 16) = 200.7]. It was representative of at least three independent experiments. The data were presented as mean ± standard error of the mean.

In addition, we constructed a plasmid encoding the SARS-CoV-2 nsp12/RdRp human codon-optimized sequence and tagged an N-terminal or C-terminal Flag to compare whether the N-term or C-term Flag tag could interrupt the RNA polymerase activity. At first, we confirmed the production of RdRp protein by the transfection of pCI-SARS2-nsp12-N-term Flag (pCI-SARS2 nsp12N) and pCI-SARS2-nsp12-C-term Flag (pCI-SARS2 nsp12C) using western blotting with an anti-Flag antibody (Figure 2A). pCI-SARS2-nsp12N or pCI-SARS2-nsp12C were transfected dose-dependently with the p(+)FLuc-(−)UTR-NLuc reporter plasmid to test the functional activity of C-term or N-term Flag-tagged SARS-CoV-2 RdRp. We found that the NLuc values were dose-dependently enhanced by the increasing concentrations of pCI-SARS2-nsp12N or pCI-SARS2-nsp12C. The relative NLuc values of the cells expressing SARS nsp12N and nsp12C were comparable, suggesting that the Flag tag did not disrupt the SARS-CoV-2 RdRp activity (Figure 2B). The relative NLuc value of the cells expressing SARS2-nsp12N was 1.1-fold higher than that of the cells expressing SARS2-nsp12C, at 2.7 and 2.4-fold at 120 ng of plasmid, respectively. So, we selected pCI-SARS2 nsp12N for further study.

We tested the system with the other SARS-CoV-2 viral protein, nsp5/3Clpro protease, to verify the specificity of the p(+)FLuc-(−)UTR-NLuc reporter plasmid to SARS-CoV-2 RdRp activity. We observed no induction of the relative NLuc value by the 3Clpro protease (Figure 2C). We also validated the p(+)FLuc-(−)UTR-NLuc reporter plasmid with polyA_33_ in 3′-UTR region [p(+)FLuc-(−)UTR-NLuc-A_33_], because the 3′-UTR of SARS-CoV-2 was attached with polyA_33_, which stabilizes RNA, stimulates translation and was used as a template for the generating negative-sense RNA [4,14]. However, when we compared the (+)FLuc-(−)UTR-NLuc reporter vector with polyA_33_ at 3′-UTR to that without the polyA_33_, we observed that the relative NLuc activity was decreased with p(+)FLuc-(−)UTR-NLuc with polyA_33_ compared to p(+)FLuc-(−)UTR-NLuc without polyA_33_, at 1.3 and 2.6-fold at 120 ng of plasmid, respectively (Figure 2D). Therefore, we used the p(+)FLuc-(−)UTR-NLuc reporter plasmid without polyA_33_ and pCI-SARS-CoV-2 nsp12N for the cell-based SARS-CoV-2 RdRp activity assay system.

### 3.2. Effect of Accessory Proteins nsp7 and nsp8 SARS-CoV-2 RdRp Activity

**Figure 3:**
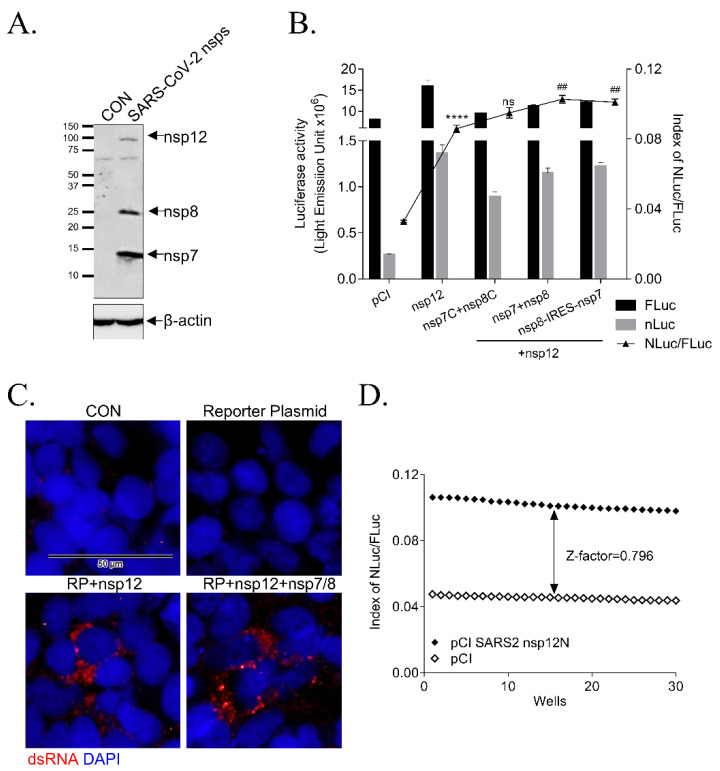
Effect of SARS-CoV-2 nsp7 and nsp8 on RdRp activity. (**A**) The expression of nsp7, nsp8 and nsp12 genes was detected with an anti-Flag antibody using Western blotting 24 h after transfecting HEK293 T cells with pCI (CON), pCI-SARS2-nsp12N, pCI-SARS2-nsp7C, or pCI-SARS2-nsp8C (SARS-CoV-2 nsps). (**B**) A cell-based SRAS-CoV-2 RdRp activity assay with the empty pCI plasmid (pCI), pCI-SARS2-nsp12N (nsp12), or pCI-SARS2-nsp12N and 3 types of nsp7 and nsp8 gene expression plasmids, C-terminal Flag-tagged (nsp7C + nsp8C), no Flag tag (nsp7 + nsp8), or pCI-SARS2-nsp8-IRES-nsp7 (nsp8-IRES-nsp7) and the p(+)FLuc-(−)UTR-NLuc reporter plasmid at the ratio of 20:120:20:20 (reporter plasmid:nsp12N:nsp7:nsp8) or 20:120:40 (reporter plasmid:nsp12N:nsp8-IRES-nsp7). FLuc and NLuc activities were measured and NLuc/FLuc values were graphed (*n* = 3; **** *p* < 0.0001, nsp12 effect, F(4, 10) = 190.8, vs. pCI; ## *p* < 0.01, nsp7 and nsp8 effect, F(3, 8) = 10.71, vs. nsp12). (**C**) Immunofluorescence staining with anti-dsRNA-specific K1 antibody (red) and DAPI (blue) 24 h after transfection with the p(+)FLuc-(−)UTR-NLuc reporter plasmid (RP) and pCI-SARS2 nsp12N (nsp12) with or without pCI-SARS2 nsp7C and pCI-SARS2 nsp8C (nsp7/8). The scale bar: 50 μm. (**D**) Z-factor calculation using Zhang’s formula. The HEK293T cells seeded in a 96-well plate were transfected with p(+)FLuc-(−)UTR-NLuc and pCI or pCI-SARS2-nsp12N. After 24 h, we measured the FLuc and NLuc activities and the Z-factor between the negative (pCI) and positive groups (pCI-SARS2-nsp12N) was calculated to be 0.796. It was representative of at least three independent experiments. The data were presented as mean ± standard error of the mean.

Among the SARS-CoV viral proteins, nsp7 and nsp8 have been reported as co-factors of RdRp [15,16,17,18]. Therefore, we tested the effect of nsp7 and nsp8 proteins on RdRp activity in this cell-based assay system. We generated the following three types of plasmids expressing the nsp7 and nsp8 genes: plasmids with a C-term Flag tag (pCI-SARS2-nsp7C and pCI-SARS2-nsp8C) or without Flag tag (pCI-SARS2-nsp7 and pCI-SARS2-nsp8) and a plasmid containing the internal ribosome entry site (IRES) between nsp8 and nsp7 (pCI-SARS2-nsp8-IRES-nsp7). The encoded proteins, C-term Flag tag nsp7 protein at 10 kDa, C-term Flag tag nsp8 protein at 22 kDa and N-term Flag tag nsp12 at 102 kDa, were detected in the HEK293T cells after transfection with pCI-SARS2-nsp7C, pCI-SARS2-nsp8C and pCI-SARS-nsp12N using western blotting (Figure 3A).

We transfected three types of nsp7 and nsp8 expression plasmids at a ratio of the 20:120:20:20 ng (reporter: nsp12: nsp8: nsp7 plasmid) or 20:120:40 ng (reporter: nsp12: pCI-SARS2-nsp8-IRES-nsp7) in HEK293T cells and detected SARS-CoV-2 RdRp activity (Figure 3B). The expression of these accessory proteins without Flag tag slightly increased RdRp activity 2.6-fold by nsp12, 3.1-fold by nsp12 with nsp7/8 and 3.0-fold by nsp12 with nsp8-IRES-7. However, RdRp activity without nsp7 and nsp8 proteins was sufficiently detectable; the nsp7 and nsp8 proteins only enhanced RdRp activity slightly. Thus, we selected the pCI-SARS2-nsp12N and p(+)FLuc-(−)UTR-NLuc reporter plasmids without pCI-SARS-nsp7 and pCI-SARS-nps8 for the cell-based system.

### 3.3. Validation of the Cell-Based SARS-CoV-2 RdRp Activity Assay System

We verified the activity of SARS-CoV-2 RdRp by detecting the dsRNA replicates generated by SARS-CoV-2 RdRp using immunostaining with a dsRNA-specific antibody. The double-stranded SARS-CoV-2 RdRp replicates were formed as the foci at the peri-nuclear region in the cells transfected with pCI-SARS2-nsp12N and the reporter plasmid with or without the plasmid encoding nsp7 and nsp8 (Figure 3C). Moreover, we verified this cell-based SARS-CoV-2 RdRp activity assay system by calculating the Z-factor, an index of reproducibility and reliability of the HTS system [13] (Figure 3D). The Z-factor was calculated to be 0.798, indicating that this system was reproducible and reliable for the HTS screening of SARS-CoV-2 RdRp inhibitors.

### 3.4. Inhibition of SARS-CoV-2 RdRp Activity by Remdesivir and Lycorine

**Figure 4:**
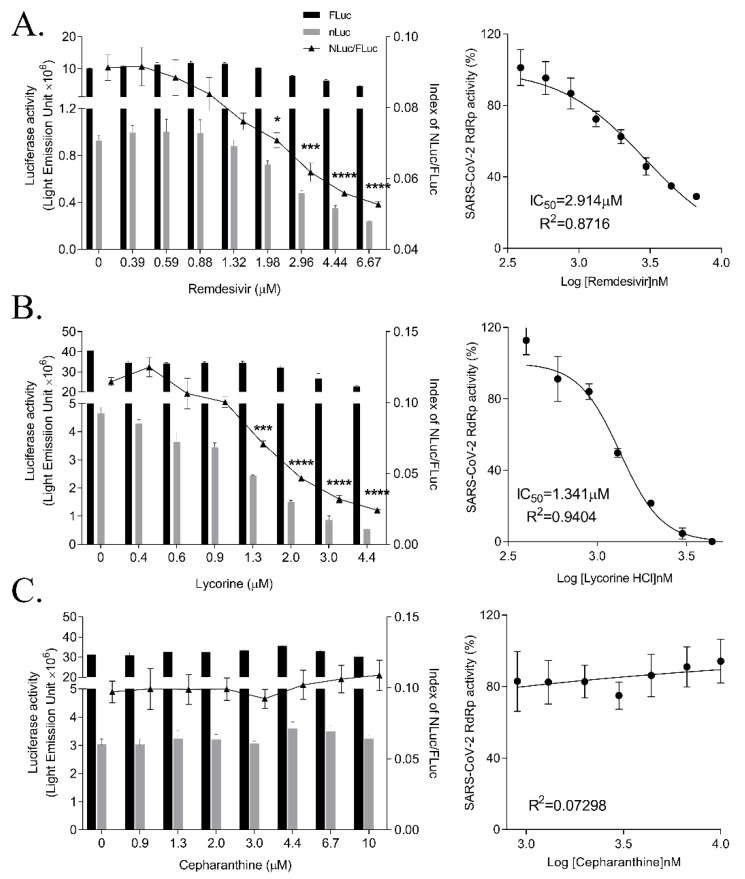
Effect of remdesivir, lycorine and cepharanthine on SARS-CoV-2 RdRp activity. HEK293T cells were transfected with p(+)FLuc-(−)UTR-NLuc and pCI-SARS2-nsp12N. After 6 h, the cells were treated with serially diluted- remdesivir (**A**), lycorine (**B**), or cepharanthine (**C**) for 15 h. FLuc and NLuc activities were measured and NLuc/FLuc ratios were determined. Statistical comparisons were conducted using one-way analysis of variance (ANOVA) followed by Bonferroni’s multiple comparison test. * *p* < 0.05; *** *p* < 0.001; **** *p* < 0.0001 vs. 0 μM (left graph). The IC_50_ values were calculated using non-linear regression analysis (right graph). The data were representative of at least three independent experiments and presented as mean ± standard error of the mean.

We further verified the cell-based system using remdesivir. Remdesivir is an adenosine analog that inhibits RdRp activity and coronavirus infection; it is FDA-approved for treating COVID-19 patients. Thus, it can be used as a positive control of the inhibition of RdRp activity [19,20]. When we treated the cells transfected with pCI-SARS2-nsp12N and the p(+)FLuc-(−)UTR-NLuc reporter plasmid with remdesivir at the indicated concentrations, the activity of SARS-CoV-2 RdRp, indicated by the relative Nano-luciferase activity, was decreased in a dose-dependent manner. Meanwhile, the activity of FLuc, acting as an internal control, was maintained. Thus, the IC_50_ of remdesivir was calculated to be 2.585 ± 0.273 μM (Figure 4A and Table 1).

Recently, we reported a natural alkaloid, lycorine, as a broad-spectrum inhibitor of coronavirus infections and a MERS-CoV RdRp inhibitor, using a cell-based MERS-CoV RdRp assay system [21]. We examined whether lycorine could inhibit SARS-CoV-2 RdRp activity by treating the cells transfected with p(+)FLuc-(−)UTR-NLuc and pCI-SARS2-nsp12N for 15 h with a lycorine and measured the SARS-CoV-2 RdRp activity. Lycorine dose-dependently reduced the NLuc activity, whereas the FLuc value remained unchanged. Lycorine completely inhibited SARS-CoV-2 RdRp activity at 4.4 μM and the IC_50_ was calculated to be 1.465 ± 0.033 μM, suggesting that lycorine inhibited SARS-CoV-2 RdRp activity more effectively than remdesivir (Figure 4B).

We tested another natural compound, cepharanthine, which was also reported to inhibit HCoV-OC43 and SARS-CoV-2 coronavirus infections [22,23]. Cepharanthine was recently suggested by virtual screening to bind at the interface active pockets of SARS-CoV-2 RdRp, nsp7 and nsp8 [24]. So, we examined the effects of cepharanthine on SARS-CoV-2 RdRp activity with or without nsp7 and nsp8 using this system. Cepharanthine treatment did not affect the activity of SARS-CoV-2 RdRp in this cell-based system (Figure 4C and Supplementary Figure S1). Therefore, we confirmed the inhibitory effects of remdesivir and lycorine, but not cepharanthine, on SARS-CoV-2 RdRp activity by the cell-based SARS-CoV-2 RdRp activity assay system.

### 3.5. Inhibition of SARS-CoV RdRp Activity by Nucleos(t)ide

**Figure 5:**
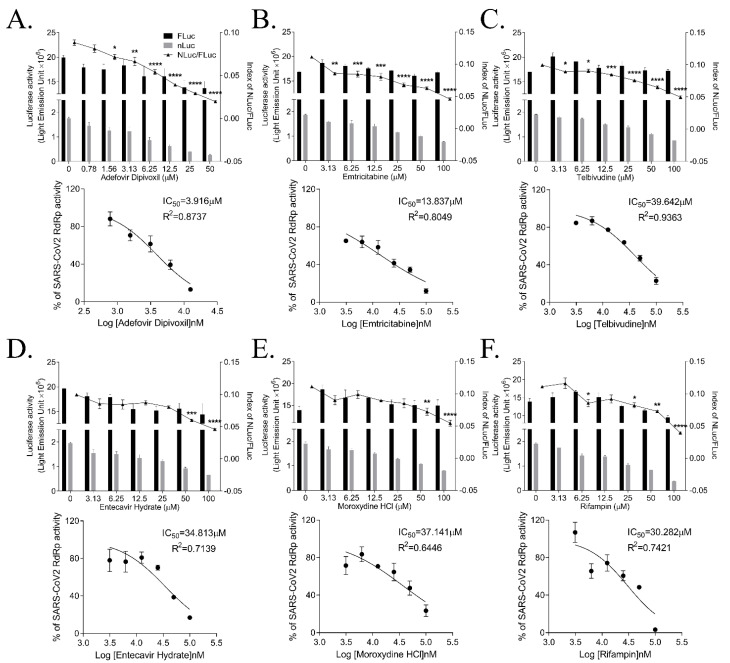
Effect of nucleoside and nucleotide analogs on SARS-CoV-2 RdRp activity. HEK293T cells were transfected with p(+)FLuc-(−)UTR-NLuc and pCI-SARS2-nsp12N. After 6 h, the cells were treated with serially diluted-adefovird dipivoxil (**A**), emtricitabine (**B**), telbivudine (**C**), entecavir hydrate (**D**), moroxydine HCl (**E**), or rifampin (**F**) for 15 h. FLuc and NLuc activities were measured to determine the NLuc/FLuc ratios. Statistical comparisons were conducted using one-way analysis of variance (ANOVA) followed by Bonferroni’s multiple comparison test. * *p* < 0.05; ** *p* < 0.01; *** *p* < 0.001; **** *p* < 0.0001 vs. 0 μM (upper graph). The IC_50_ values were calculated using non-linear regression analysis (lower graph). The data were representative of at least three independent experiments and presented as mean ± standard error of the mean.

We screened nucleos(t)ide analogs using the cell-based SARS-CoV-2 RdRp activity assay system to identify inhibitors of SARS-CoV-2 RdRp activity. Adefovir dipivoxil is a nucleoside analog inhibiting the reverse transcriptase activity of HBV; it is FDA-approved for chronic hepatitis B [25]. When we applied adefovir dipivoxil to our cell-based system, it dose-dependently reduced SARS-CoV-2 RdRp activity with an IC_50_ value of 3.785 ± 0.866 μM (Figure 5A). Its inhibitory effect on SARS-CoV RdRp activity was comparable to that of remdesivir.

Emtricitabine is known to inhibit the activity of human immunodeficiency virus reverse transcriptase via its incorporation into the DNA, terminating the DNA chain elongation [26]. We found that emtricitabine effectively inhibited SARS-CoV-2 RdRp activity with the IC_50_ value of 15.375 ± 3.602 μM in this assay system (Figure 5B).

We have also identified moderate inhibitors of SARS-CoV-2 RdRp activity. Telbivudine, a pyrimidine 2′-deoxyribonucleoside acting as a thymidine analog to inhibit HBV DNA replication, reduced SARS-CoV-2 RdRp activity with an IC_50_ value of 45.928 ± 3.859 μM (Figure 5C). Entecavir hydrate, a guanosine analog possessing the anti-HBV activity [27], exhibited an IC_50_ value of 41.993 ± 4.162 μM (Figure 5D). Moroxydine, developed as the inhibitor of RNA or DNA viruses, including an influenza virus and HSV [28], inhibited SARS-CoV-2 RdRp with an IC_50_ of 48.929 ± 14.370 μM (Figure 5E). In addition, rifampin, also known as rifampicin and an antibiotic for tuberculosis [29], displayed an inhibitory effect on SARS-CoV-2 RdRp activity with an IC_50_ of 49.434 ± 4.020 μM (Figure 5F). Therefore, our cell-based SARS-CoV-2 RdRp activity assay system has identified these nucleos(t)ide analogs as inhibitors of SARS-CoV-2 RdRp activity, among them, adefovir dipivoxil was the most effective inhibitor.

## 4. Discussion

We have created a cell-based SARS-CoV-2 RdRp activity assay system by altering the cell-based MERS-CoV RdRp activity assay system [12]. The system consists of a bicistronic p(+)FLuc-(−)UTR-NLuc reporter plasmid and the pCI-SARS2-nsp12N plasmid. The NLuc activity of the cells transfected with the plasmids represented the RdRp activity and FLuc activity was used as the internal control. We have used this system to screen and discover inhibitors of SARS-CoV-2 RdRp.

We examined the effect of the Flag tag on RdRp activity by comparing its activity with that of the C-term Flag-tagged RdRp and N-term Flag-tagged RdRp. The N-terminal region of RdRp is important for protein folding [30,31]. However, the activities of these tagged proteins were comparable. Therefore, we chose the N-term Flag-tagged RdRp for this study due to the 10% higher activity. These results were consistent with the observation that the activity of N-term Flag-tagged MERS RdRp was higher than C-term Flag-tagged RdRp in the cell-based MERS-CoV RdRp assay system [12].

In addition, we tested the p(+)FLuc-(−)UTR-NLuc reporter plasmid with polyA_33_ or without polyA_33_ under the 3′-UTR of SARS-CoV. Although PolyA_33_ stabilizes RNA, stimulates translation and is used as a template for generating negative-sense RNA [32], we unexpectedly found that RdRp activity was decreased in the cells transfected with p(+)FLuc-(−)UTR-NLuc with polyA_33_ compared to p(+)FLuc-(−)UTR-NLuc without polyA33. Therefore, we used the reporter plasmid without polyA_33_ for a cell-based SARS-CoV-2 RdRp activity assay system.

Two nsp8, one nsp7 and one nsp12 protein of SARS-CoV-2 form the active RdRp complex with template-primer RNA and co-factors nsp8 and nsp7 proteins were reported to confer the processivity of RdRp [9,15,17,18]. We tested the effect of nsp7 and nsp8 on RdRp activity in our cell-based system. In this system, the RdRp activity without nsp7 and nsp8 proteins was already detectable and the nsp7 and nsp8 proteins only increased RdRp activity slightly. Thus, we conducted the cell-based SARS-CoV-2 RdRp activity assay system without using the plasmids encoding nsp7 and nsp8 to screen the inhibitors by targeting the nsp12 function only. Then, we should define the effect of inhibitors on the interaction and efficacy of RdRp complex with nsp7 and nsp8 proteins in more detail.

Single-strand RNA viruses replicate their RNA in the cytoplasm of the infected cells and the corresponding dsRNA foci have been detected at the peri-nuclear region by immunofluorescence staining [33]. Here, we also visualized the peri-nuclear foci in our cell-based SARS-CoV-2 RdRp activity assay system, confirming that RdRp in the system could generate p(+)FLuc-(−)UTR-NLuc reporter plasmid-originated dsRNA replicates. Moreover, the high Z-factor of this assay system confirmed its reliability and reproducibility for the SARS-CoV-2 RdRp inhibitor screening HTS system.

We validated our cell-based SARS-CoV-2 RdRp activity assay system by testing various drugs that have been effective against coronaviruses. Remdesivir is an FDA-approved first-class drug for COVID-19 and an adenosine analog inhibitor of SARS-CoV-2 RdRp [9,10]. In our system, remdesivir dose-dependently inhibited the RdRp activity. In addition, we tested lycorine, a natural alkaloid and non-nucleoside inhibitor of MERS-CoV RdRp and in silico inhibitor of SARS-CoV RdRp [21]. In our system, lycorine inhibited the activity of SARS-CoV RdRp dose-dependently, suggesting that lycorine was a more effective SARS-CoV RdRp inhibitor than remdesivir. These data were consistent with the findings that lycorine more effectively inhibited SARS-CoV-2 infection at the IC_50_ value of 0.878 ± 0.022 μM compared with remdesivir at the IC_50_ of 6.499 ± 0.256 μM. Moreover, the binding affinity of lycorine to SARS-CoV-2 RdRp at −6.2 kcal/mol is stronger than that of remdesivir at −4.7 kcal/mol [21]. Cepharanthine inhibits coronavirus infections by blocking the Ca^2+^-permeable channels during virus entry [22,23,34]. Recently, it was also suggested that cepharanthine may also bind to the interface active pockets of the SARS-CoV-2 nsp12-nsp7 and nsp12-nsp8 by virtual screening [24]. We tested cepharanthine in our assay system, but it did not display any inhibitory effect on SARS-CoV-2 RdRp activity with or without nsp7 and nsp8.

Finally, we tested the nucleos(t)ide analogs using our system to discover other inhibitors of SARS-CoV-2 RdRp. We found that the adefovir dipivoxil for treatment of HBV infection [25] effectively inhibited SARS-CoV-2 RdRp activity at a level comparable to remdesivir. The other HBV inhibitors, telbivudine [35] and entecavir hydrate [27,36], inhibited SARS-CoV-2 RdRp activity, although at a lower inhibitory ability level than adefovir dipivoxil. A nucleoside reverse transcriptase inhibitor of HIV, emtricitabine [37], also inhibited SARS-CoV-2 RdRp activity. In addition, we found that moroxydine, a broad antiviral agent against DNA and RNA viruses [28] and rifampin, a macrocyclic antibiotic for tuberculosis [38], repressed SARS-CoV-2 RdRp activity moderately. Thus, we have identified six nucleos(t)ide/ analogs as SARS-CoV-2 RdRp inhibitors using the cell-based SARS-CoV-2 RdRp activity assay system. Among them, adefovir dipivoxil was likely the strongest inhibitor comparable to the already-reported RdRp inhibitors, remdesivir and lycorine.

In summary, we have established a cell-based SARS-CoV-2 RdRp activity assay system to screen the inhibitor of SARS-CoV-2 RdRp. We confirmed the inhibitory activity of remdesivir and lycorine on SARS-CoV-2 RdRp using this system. In addition, we screened the nucleos(t)ide analogs and identified six nucleos(t)ide analogs as novel SARS-CoV-2 RdRp inhibitors and therapeutic candidates for the COVID-19. Thus, our assay system can provide an effective HTS platform for developing prophylactic and therapeutic drugs for COVID-19 and other emerging coronavirus infections.

## Figures and Tables

**Table 1:** IC_50_ and inhibition percentage of SARS-CoV-2 RNA-dependent RNA polymerase (RdRp) activity at the maximum concentrations. It was representative of at least three independent experiments. The data were presented as mean ± standard error of the mean.

Name	IC_50_ (μM)	Max. Dose (μM)	Inhibition % of RdRp Activity at Max. Dose
Remdesivir	2.585 ± 0.273	6.7	71.03
Lycorine	1.465 ± 0.033	4.4	100
Cepharanthine	>10	10	5.84
Adefovir Dipivoxil	3.785 ± 0.866	12.5	86.98
Emtricitabine	15.375 ± 3.602	100	88.21
Telbivudine	45.928 ± 3.859	100	76.92
Entecavir Hydrate	41.993 ± 4.162	100	83.09
Moroxydine	48.929 ± 14.370	100	76.54
Rifampin	49.434 ± 4.020	100	96.73

## Data Availability

Not applicable.

## Supplementary Materials

The following are available online at https://www.mdpi.com/article/10.3390/biomedicines9080996/s1, Figure S1: Effects of cepharanthine on SARS-CoV-2 RdRp activity in the cell-based activity assay with nsp7 and nsp8.

## Abbreviations

3CLpro	3C-like protease
CoV	coronavirus
COVID-19	coronavirus disease 2019
C-term	C-terminal
FDA	Food and Drug Administration
FLuc	firefly luciferase
HBV	hepatitis B virus
HDV	hepatitis delta virus
HIV	human immunodeficiency virus
HSV	herpes simplex virus
HTS	high-throughput screening
IRES	internal ribosome entry site
MERS	Middle East respiratory syndrome
NLuc	Nano-glo^®^ luciferase
NSP	Nonstructural proteins
NTP	Nucleoside triphosphate
N-term	N-terminal
ORF	open reading frame
PLpro	papain-like protease
RdRp	RNA-dependent RNA polymerase
SARS	severe acute respiratory syndrome
SARS-CoV-2	severe acute respiratory syndrome coronavirus 2
UTR	untranslated region
