# Supplementary material for: SARS-CoV-2 RdRp Inhibitors Selected from a Cell-Based SARS-CoV-2 RdRp Activity Assay System
Source: Biomedicines. 2021 Aug 11;9(8):996. doi: 10.3390/biomedicines9080996 (PMC8392292; doi:10.3390/biomedicines9080996)
Supplement: Supplementary file 1 [file biomedicines-09-00996-s001.zip › biomedicines-1326042-supplementary.pdf]

Supplementary Figure S1.

A.

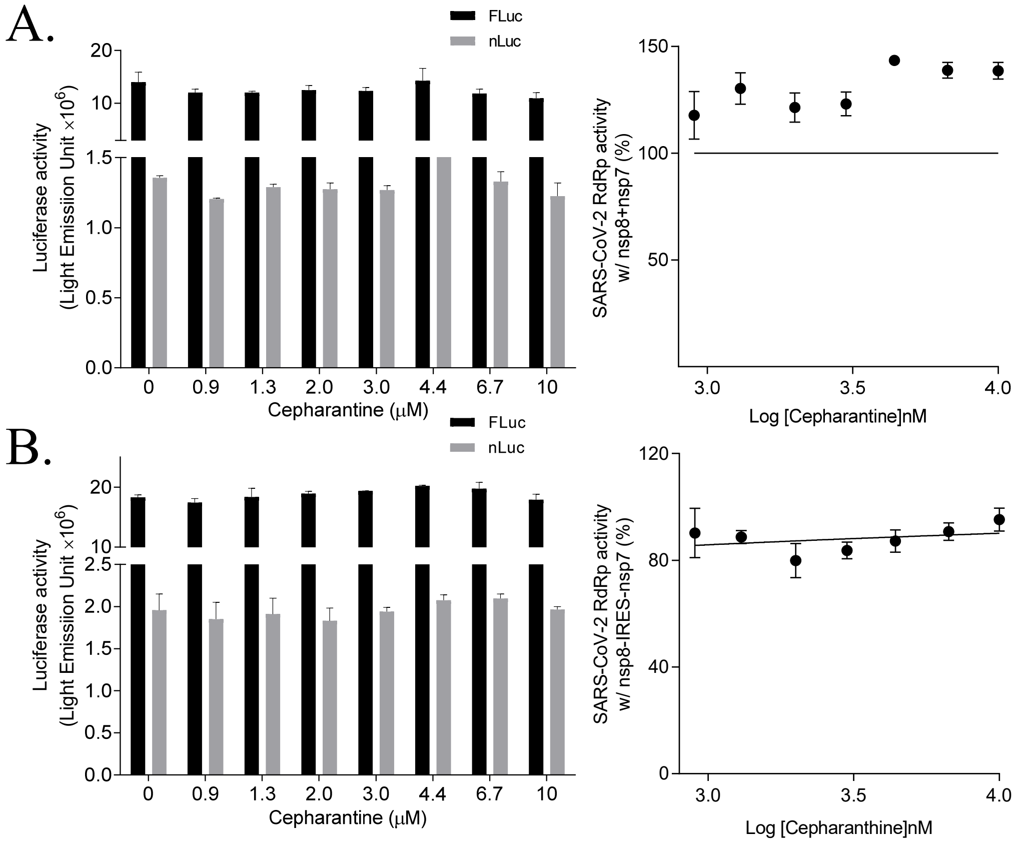

Supplementary Figure S1. Effects of cepharanthine on SARS-CoV-2 RdRp activity in the cell-based activity assay with nsp7 and nsp8. A cell-based SRAS-CoV-2 RdRp activity assay with pCI-SARS2-nsp12N and plasmids expressing nsp7 and nsp8 genes without Flag tag (nsp7 + nsp8) (a) or the nsp8-IRES-nsp7 plasmid (b).
